# Vaspin Attenuates Atrial Abnormalities by Promoting ULK1/FUNDC1-Mediated Mitophagy

**DOI:** 10.1155/2022/3187463

**Published:** 2022-11-15

**Authors:** Yanmin Zhu, Zhoushan Gu, Jiayu Shi, Chu Chen, Haixia Xu, Qi Lu

**Affiliations:** ^1^Department of Cardiology, Affiliated Hospital of Nantong University, Jiangsu 226001, China; ^2^Department of Cardiology, The First Peoples' Hospital of Taicang, The Affiliated Taicang Hospital of Soochow University, Taicang, Jiangshu 215300, China

## Abstract

The worldwide incidence and prevalence of atrial fibrillation (AF) are increasing, making it a life-threatening condition due to the higher numbers of people suffering from obesity. Vaspin, an adipokine derived from epicardial adipose tissue, has been reported to reduce inflammation, inhibit apoptosis, and induce autophagy; however, its role in the pathogenesis of AF is not known. In this study, we investigated the role of vaspin in patients with AF and explored the molecular mechanisms using atrial myocytes *in vitro*. Our data showed that vaspin levels were significantly reduced in the plasma of patients with AF. Lower plasma levels of vaspin were also associated with a higher risk of AF in patients with obesity. Vaspin treatment *in vitro* alleviated cardiomyocyte injury, atrial fibrosis, atrial myocyte apoptosis, and mitochondrial injury in atrial myocytes following Ang-II stress. Moreover, our results demonstrated that vaspin protected against Ang-II-induced atrial myocyte dysfunction by inducing mitophagy. We also observed that vaspin treatment enhanced the phosphorylation of Fun14 domain-containing protein 1 (FUNDC1) at Ser17 by unc-51 like autophagy activating kinase 1 (ULK1), resulting in the induction of mitophagy. These positive effects of vaspin were reversed by ULK1 silencing in Ang-II-stimulated HL-1 cells. Our study is the first to propose that vaspin plays a vital role in AF pathogenesis via ULK1/FUNDC1-regulated mitophagy and could be a novel therapeutic target for AF.

## 1. Introduction

Atrial fibrillation (AF), one of the most common types of arrhythmias, is a global health care problem [1]. Patients with AF suffer from severe symptoms, as well as from an increased risk of stroke and mortality [2]. Atrial fibrosis has emerged as a hallmark of atrial remodeling during AF development [3, 4]. Angiotensin ІІ (Ang-ІІ), a peptide hormone that plays a key role in the renin-angiotensin system (RAS), induces mitochondrial reactive oxygen species (ROS) generation, which, in turn, activates numerous signaling molecules, such as proinflammatory cytokines, leading to cardiac remodeling and atrial fibrosis during AF [3, 5, 6]. The pathophysiology of AF and atrial fibrosis is a combination of complex processes, and the precise molecular mechanisms underlying AF development have not yet been elucidated.

Several studies have indicated that obesity is a major modifiable risk factor for AF [7, 8]. Although body mass index (BMI) and other clinical measures are key indicators of general adiposity, it has been proposed that epicardial adipose tissue (EAT) surrounding the heart could be a more accurate parameter, since there is a positive correlation between adipose tissue and AF [9]. Adipokines derived from EAT can act directly on the connected atrial myocardium, thus facilitating the process of atrial remodeling [10, 11]. It has been reported that adipokine activin A, matrix metalloproteinase 2 (MMP2), and transforming growth factor-*β*1 (TGF-*β*1) overexpression contribute to atrial fibrosis and AF, further supporting this hypothesis [12].

Visceral adipose tissue-derived serine protease inhibitor (vaspin), a 392–395 amino acid protein, has been identified as a compensatory adipokine that improves insulin sensitivity and anti-inflammatory response in obesity, as well as its metabolic consequences [13, 14]. Furthermore, several studies have suggested that decreased vaspin plasma levels are associated with the progression of cardiovascular disease [15]. It has been previously reported that vaspin prevents an increase in blood pressure by inhibiting oxidative and inflammatory responses in peripheral vascular cells [16]. Furthermore, vaspin could be used as a prognostic biomarker in patients with acute myocardial infarction [17]. Previous studies have also suggested that vaspin suppresses apoptosis and protects against vascular injury in diabetes by binding to the glucose-regulated protein 78 (GRP78)/voltage-dependent anion channel (VDAC) complex [18, 19]. However, the role of vaspin, as well as its mechanism of action in atrial fibrosis and AF, is not clear.

Mitochondrial dysfunction has been implicated in the progression of AF [20, 21]. Mitophagy is an essential intracellular mechanism involved in the regulation of mitochondrial quality control by selectively removing dysfunctional or unwanted mitochondria [22–25]. It has been previously reported that mitophagy impairment plays a crucial role in the atrial myocytes of patients with AF [26]. Fun14 domain-containing protein 1 (FUNDC1) has been identified as a new mitophagic receptor, which binds to microtubule-associated protein light chain 3 beta (LC3B) [27, 28]. Furthermore, unc-51 like autophagy activating kinase 1 (ULK1) induces the phosphorylation of FUNDC1 at Ser17, promoting its direct interaction with LC3B and mitophagic activity in response to mitochondrial damage [29]. However, the role of vaspin in mitophagy and atrial fibrosis remains unclear. In this study, we investigated the role of vaspin in mitochondrial integrity and atrial myocyte dysfunction in AF and atrial fibrosis, as well as the underlying molecular mechanism(s) involved in ULK1/FUNDC1-mediated mitophagy.

## 2. Materials and Methods

### 2.1. Human Subjects

Patients with obesity (*n* = 90) receiving radiofrequency ablation for AF from the Department of Cardiology of the Affiliated Hospital of Nantong University between January 2016 and June 2018 were recruited for the study, and patients with obesity and sinus rhythm (SR) (*n* = 73) were enrolled as controls. The inclusion criteria were as follows: (1) patients aged between 18 and 80 years with a BMI > 24 and (2) AF diagnosed using 12-lead electrocardiography. Patients with structural heart diseases, infectious diseases, cancer, and severe hepatic or renal dysfunction were excluded. This study was approved by the Institutional Ethics Committee of the Affiliated Hospital of Nantong University (2019-K071). Blood samples from all patients were collected into the test tubes containing ethylenediaminetetraacetic acid (EDTA) and centrifuged at 3,000 revolutions per minute (rpm) for 15 min. Plasma was collected and frozen at − 80 °C.

### 2.2. Enzyme-Linked Immunosorbent Assay (ELISA)

Vaspin, MMP2, and TGF-*β* levels in plasma samples were detected using ELISA kits (Enzyme-linked Biotechnology Corporation, Shanghai, China), according to the manufacturer's instructions.

### 2.3. Atrial Myocyte Culture and siRNA Transfection

HL-1 cells (mouse atrial myocytes) were cultured in Claycomb medium supplemented with 10% fetal bovine serum (FBS, Gibco, USA), norepinephrine (100 *μ*M, Sigma-Aldrich, MO, USA), and L-glutamine (2 mM, Gibco, USA). Next, cells were treated with Ang-II (1 *μ*M, Sigma, USA) for 24 h as previously described [30, 31]. For silencing experiments, cells were cultured and transfected with scrambled siRNA (control) or ULK1 siRNA (5′-AAGGACCGCAUGGACUUUGAU-3′) for 48 h using Lipofectamine 3000 transfection reagent.

### 2.4. Cell Viability Assay

Cell viability was evaluated using the 3-(4,5)-dimethylthiahiazo(-z-y1)-3,5-diphenytetrazoliumromide (MTT) assay kit (Beyotime, Shanghai, China) according to the manufacturer's protocol. Briefly, the MTT working solution was added to the wells, and cells were cultured for 4 h. Next, MTT formazan was dissolved in dimethyl sulfoxide (DMSO), and the absorbance was measured at 490 nm.

### 2.5. The Terminal Deoxynucleotidyl Transferase (TdT)-Mediated dUTP Nick-End Labeling (TUNEL) Assay

Cardiomyocyte apoptosis was evaluated as previous described [32]. Briefly, cells were permeabilized and treated with TUNEL reaction mix (Roche, Mannheim, Germany) for at least 1 h at 37 °C. The number of positive cells/total number of cells was used to calculate the percentage of TUNEL-positive cells (the apoptosis index).

### 2.6. Mitochondrial Membrane Potential (MMP) and ROS Evaluation

MMP was evaluated using a JC-1 kit (Beyotime, Shanghai, China) and tetramethylrhodamine methyl ester (TMRM) staining (Thermo Fisher Scientific, MA). Briefly, cells were cultured with 5 *μ*M JC-1 working dye at 37 °C for 30 min. MMP was quantified as the ratio of red to green fluorescence intensity. For TMRM staining, the TMRM solution was added to cells for 30 min at 37 °C. ROS production in cells was detected using 2′,7′-dichlorofuorescein-diacetate (DCFH-DA; Beyotime, Shanghai, China) and MitoSOX (Thermo Fisher Scientific, MA, USA). After Ang-II and vaspin treatment, cells were incubated with 10 *μ*M DCFH-DA or 1 *μ*M MitoSOX in FBS-free DMEM for 10 min. Images were acquired using a laser confocal microscope (Leica, Germany).

### 2.7. Autophagy and Mitophagy Evaluation

HL-1 cells were plated and then transfected with GFP-RFP-LC3 adenovirus (Hanbio, Shanghai, China). GFP and mRFP fluorescent puncta were quantified and analyzed using the ImageJ software. Autophagic flux was evaluated as the number of yellow puncta (early autophagosomes) and red puncta (late autolysosomes) [32].

To evaluate mitophagy, we used mito-Keima (HanBio Technology Co. Ltd., Shanghai, China), a pH-sensitive lysosomal protease-resistant fluorescent probe, whose excitation shifts from 457 to 561 nm in the acidic environment of lysosomes [32]. Briefly, cells were transfected with mito-Keima adenoviruses (MOI = 10) for 48 h. The fluorescent images were acquired using a microscope (Leica, Germany) and then quantified using the ImageJ software.

### 2.8. Mitochondrial Fraction Preparation

Mitochondria were extracted from HL-1 cells using a mitochondrial isolation kit (ab110170, Abcam) as previously described [33]. Briefly, cells were collected into the isolation buffer and homogenized with a glass Dounce homogenizer for approximately 30 strokes. The supernatants were centrifuged at 1,000×*g* for 10 min, and the pellets were resuspended and homogenized. Next, the supernatants were centrifuged at 12,000×*g* for 15 min, and the pellets were resuspended in RIPA lysis buffer.

### 2.9. Immunofluorescence Staining

HL-1 cells were fixed, permeabilized, blocked, and incubated with specific primary antibodies overnight, followed by corresponding Alexa Fluor secondary antibodies at room temperature. Images were obtained using a laser confocal microscope (Leica, Germany).

### 2.10. Western Blot

Cells were lysed in RIPA lysis buffer containing protease and phosphatase inhibitors. Samples (25-50 *μ*g) were loaded onto 10–12% Bis-Tris gels and then transferred onto the polyvinylidene fluoride (PVDF) membranes. The membranes were blocked and incubated with primary antibodies against *β*-myosin heavy chain (*β*-MHC, 1 : 1000, ab249500), atrial natriuretic peptide (ANP, 1 : 1000, ab225844), matrix metallopeptidase-9 (MMP-9,1 : 1000, ab283575), matrix metallopeptidase-2 (MMP-2, 1 : 1000, ab92536), B-cell lymphoma 2 (Bcl-2, 1 : 1000, CST#3498), cleaved caspase-9 (1 : 1000, CST#20750), BCL-2 associated X (Bax, 1 : 1000, CST#41162), autophagy-related protein 5 (Atg5, 1 : 1000, ab108327), Beclin1 (1 : 1000, ab19662), LC3 (1 : 1000, ab192890), oxidative phosphorylation (OXPHOS, 1 : 1000, ab110413), FUNDC1 (1 : 250, ab224722), p^(Ser17)^-FUNDC1 (1 : 250), and ULK1 (1 : 1000, ab8054) overnight at 4 °C. The p^(Ser17)^-FUNDC1 antibody was produced as previously described [34]. Membranes were then incubated with secondary antibodies for 1 h. The bands observed were measured using Image Lab 3.0 (National Institutes of Health, Bethesda, USA).

### 2.11. Statistical Analysis

Data are shown as the mean ± standard error of the mean (SEM). The results were analyzed using SPSS software (version 22.0; IBM, Armonk, NY, USA) or GraphPad Prism 8.0 (GraphPad, CA, USA). Student's *t*-test was performed for two-group comparisons, and ANOVA followed by Tukey's post hoc test was performed for multiple group comparisons. Comparisons between groups for categorical clinical variables were performed using the chi-squared test. Logistic regression analyses were conducted to assess the association between plasma vaspin levels and the incidence of AF in obese patients, and the results were presented as odds ratios (ORs) with 95% confidence intervals (CIs). Receiver operating characteristic (ROC) curves were used to calculate the area under the curve (AUC). The cut-off value of vaspin for AF severity and prognosis was confirmed using Youden's index. Statistical significance was set at *p* value < 0.05.

## 3. Results

### 3.1. Vaspin Concentration Is Lower in the Serum of Patients with AF

**Figure 1:**
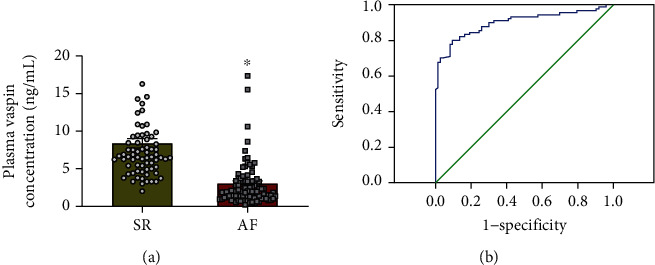
Vaspin concentration is lower in the serum of patients with atrial fibrillation (AF). (a) Plasma vaspin levels were reduced in AF patients. (b) Receiver operating characteristic (ROC) curves for the evaluation of vaspin levels in patients with AF. The area under the curve (AUC) was 0.90, with a cut-off value of 3.716 ng/mL, sensitivity of 90.4%, and specificity of 78.9%. ^∗^*p* < 0.05*vs*. sinus rhythm (SR) group.

The demographic, echocardiographic, and laboratory data of 163 patients hospitalized in the Department of Cardiology at the Affiliated Hospital of Nantong University between January 1, 2016, and June 28, 2018, were analyzed. All patients fulfilled the inclusion and exclusion criteria. As shown in Table 1, there were no significant differences between patients with AF and those with sinus rhythm (SR) in age (*p* = 0.533), sex (*p* = 0.259), BMI (*p* = 0.078), smoking status (*p* = 0.726), and drinking status (*p* = 0.157). Furthermore, no differences were observed between these two groups based on the comorbidities associated with diabetes (*p* = 0.299), hypercholesterolemia (*p* = 0.505), and hypertension (*p* = 0.335). In addition, there was no disparity in cardiac function, as determined by the left ventricular ejection fraction (LVEF). However, our data indicated that the left atrial diameter (LAD) was significantly increased in patients with AF (*p* = 0.009; Table 1). At the same time, clinical parameters, such as triglycerides (TG), total cholesterol (TC), and low-density lipoprotein-cholesterol (LDL-c), were not significantly different between these two groups. Furthermore, our results demonstrated that MMP2 and B-type natriuretic peptide (BNP) concentrations were higher in patients with AF, while vaspin expression levels in patients with AF were significantly lower than those in patients with SR (Figure 1(a)).

To investigate the association between circulating vaspin levels and AF risk, logistic regression analysis was performed (Table 2). Several variables were independently associated with AF, including LAD, BNP, vaspin, MMP2, and TGF-*β* levels. Furthermore, lower plasma levels of vaspin were associated with an increased risk of AF in patients with obesity (*p* < 0.001, OR = 1.437, and 95%CI = 1.178 − 1.753). ROC curve analysis indicated that the vaspin serum level was a risk biomarker for AF in patients with obesity: AUC for vaspin was 0.90, with a cutoff value of 3.716 ng/mL, sensitivity of 90.4%, and specificity of 78.9% (Figure 1(b)).

### 3.2. Vaspin Supplementation Partially Attenuates Ang-II-Induced Atrial Myocyte Fibrosis

**Figure 2:**
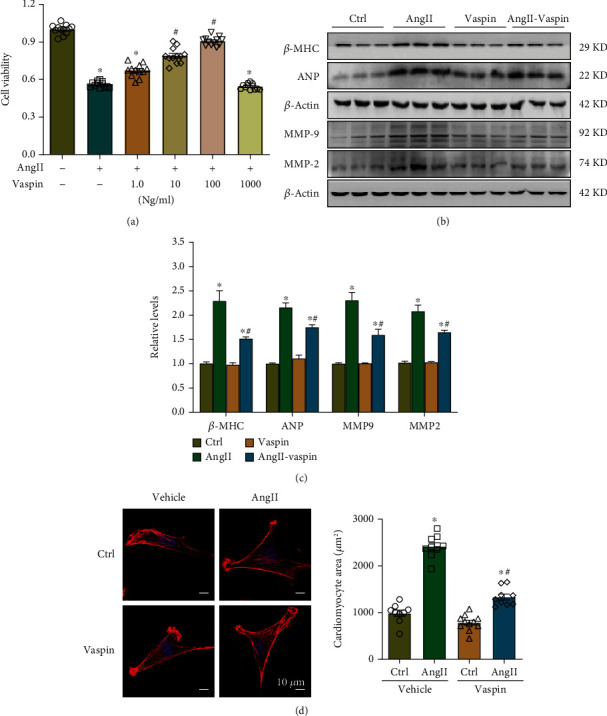
Vaspin supplementation attenuates Ang-II-induced atrial myocytes dysfunction. (a) MTT analysis revealed cell viability in Ang-II stimulated HL-1 cells treated with vaspin (1.0, 10, 100, and 1000 ng/mL), *n* = 12/group, data from three independent experiments. (b–c) Representative immunoblots and quantification of *β*-MHC, ANP, MMP-9, and MMP-2, *n* = 6/group, data from three independent experiments. (d) Representative images and quantification of phalloidin staining, scale bar = 10 *μ*m. Data are presented as the mean ± SEM, ^∗^*p* < 0.05*vs*. control group, ^#^*p* < 0.05*vs*. Ang-II group.

Several studies have demonstrated that Ang-II stimulation can lead to atrial fibrosis and AF. To evaluate the role of vaspin in atrial myocytes, cells were treated with Ang-II as previously described [30, 31]. The MTT assay showed that vaspin significantly improved cell viability in a dose-dependent manner (Figure 2(a)), similar to previous studies [35], and 100 ng/mL vaspin concentration was selected for subsequent experiments. Next, we examined the expression of *β*-MHC, ANP, MMP-2, and MMP-9 in HL-1 cells in response to Ang-II (1 *μ*M) treatment for 24 h. Our data showed that Ang-II-treated groups had higher *β*-MHC, ANP, MMP-2, and MMP-9 expression levels and this effect was partially reversed by vaspin administration (Figures 2(b)–2(c)). Furthermore, vaspin treatment significantly reduced the size of Ang-II-treated cells as determined by the phalloidin staining assay, consistent with increased *β*-MHC and ANP protein levels (Figure 2(d)). These data indicated that vaspin reversed the impaired cardiomyocyte function and atrial myocyte fibrosis in response to Ang-II stimulation.

### 3.3. Vaspin Protects against Ang-II-Induced Atrial Myocyte Apoptosis

**Figure 3:**
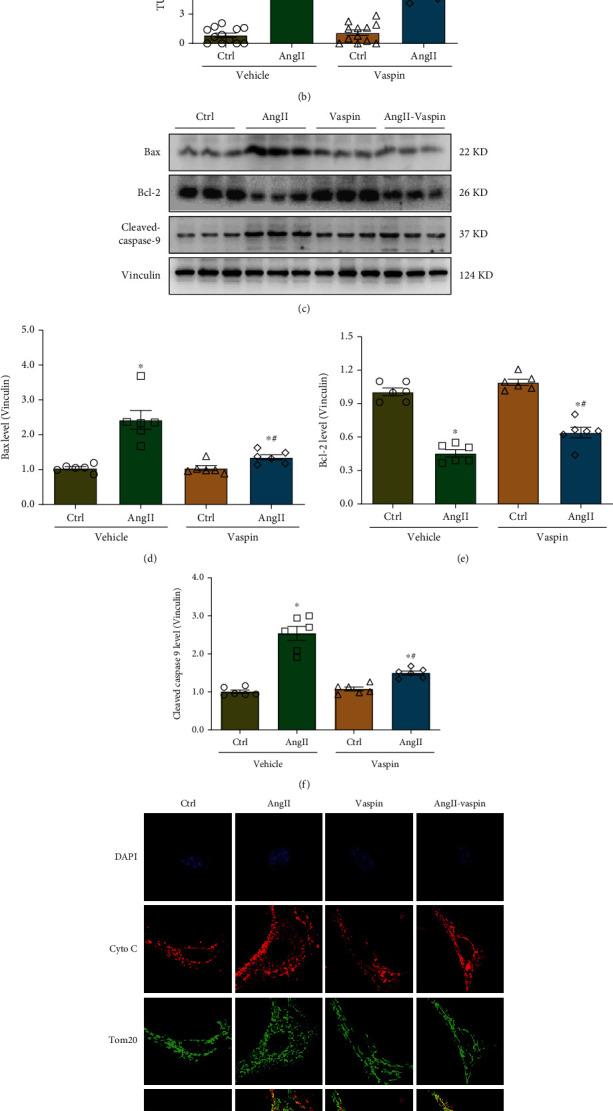
Vaspin treatment decreases Ang-II-induced apoptosis in atrial myocytes. (a–b) Representative images and quantification of TUNEL assay, *n* = 12 fields/group, data from three independent experiments. (c–f) Representative immunoblots and quantification of Bax, Bcl-2, and cleaved caspase 9 levels, *n* = 6/group. (g–h) Representative images and quantification of mitochondrial cytochrome c release, *scale* *bar* = 5 *μm*. Data are presented as the mean ± SEM, ^∗^*p* < 0.05*vs*. control group, ^#^*p* < 0.05*vs*. Ang-II group.

To investigate the effect of vaspin on atrial myocyte apoptosis after Ang-II stimulation, a TUNEL assay was performed. The results showed that Ang-II stimulation markedly induced apoptosis in HL-1 cells and this effect was partially attenuated by vaspin treatment (Figures 3(a)–3(b)). Furthermore, the levels of Bax and cleaved caspase-9 were upregulated, while the protein levels of Bcl-2 were decreased in the Ang-II group, consistent with the results of the TUNEL assay, and this effect was reversed by vaspin treatment (Figures 3(c)–3(f)). It has been reported that cytochrome c is released from damaged mitochondria, leading to the induction of apoptosis [33]. Our results indicated that Ang-II treatment triggered cytochrome c release from mitochondria and subsequently induced mitochondria-associated apoptosis in HL-1 cells, which was partially reversed by vaspin (Figures 3(g)–3(h)).

### 3.4. Vaspin Alleviates Ang-II-Induced Mitochondrial Dysfunction in HL-1 Cells

**Figure 4:**
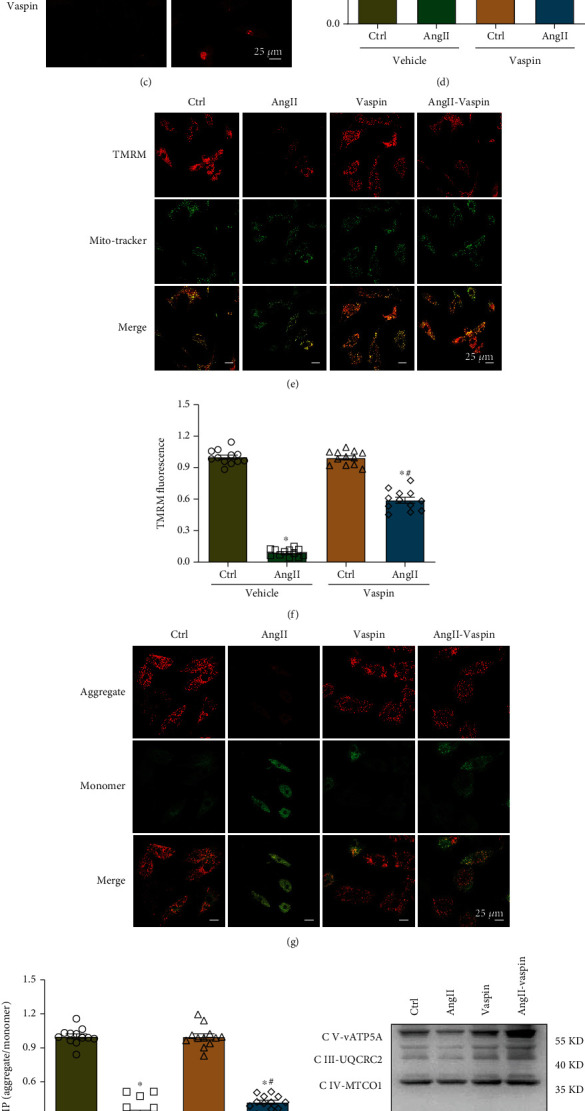
Vaspin alleviates Ang-II-induced mitochondrial dysfunction in HL-1 cells. (a–b) Representative images and quantification of reactive oxygen species (ROS) accumulation using DCFH-DA staining, scale bar = 25 *μ*m. (c–d) Representative images and quantification of mitochondrial oxidative stress using MitoSOX staining, scale bar = 25 *μ*m. (e–f) Representative images and quantification of TMRM staining, scale bar = 25 *μ*m. (g–h) Representative images and quantification of JC-1 staining, scale bar = 25 *μ*m, *n* = 12 fields/group, data from three independent experiments (panels a–h). (i–j) OXPHOS in Ang-II-stimulated HL-1 cells in the presence or absence of vaspin treatment, *n* = 6 samples/group (panels i–j). Data are presented as the mean ± SEM, ^∗^*p* < 0.05*vs*. control group, ^#^*p* < 0.05*vs*. Ang-II group.

It has been reported that vaspin treatment protects against Ang-II-induced mitochondria-associated apoptosis in atrial myocytes [20]. Therefore, we evaluated mitochondrial function using DCFH-DA, MitoSOX, TMRM, and JC-1 staining. To evaluate ROS production in the cardiomyocytes, we used DCFH-DA and MitoSOX staining. Our results showed that ROS accumulation in HL-1 cells was significantly increased in the Ang-II groups compared with that in the control groups, as demonstrated by the fluorescence intensity of DCFH-DA and MitoSOX, and this effect was diminished by vaspin treatment (Figures 4(a)–4(d)). TMRM results showed that the percentage of active mitochondria in the Ang-II group was reduced; however, this effect was significantly improved by vaspin treatment (Figures 4(e)–4(f)). Furthermore, red/green fluorescence intensity was reduced in Ang-II-stimulated cardiomyocytes, while vaspin treatment reversed this effect (Figures 4(g)–4(h)). At the same time, vaspin treatment alone did not affect TMRM and MMP levels.

To analyze mitochondrial respiratory function, mitochondrial oxidative phosphorylation (OXPHOS) was evaluated. Ang-II induced a lower level of respiratory chain components, including complexes I, II, III, IV, and V; however, this complex abundance appeared to be alleviated by vaspin (Figures 4(i)–4(j)). These data suggested that vaspin treatment reduced Ang-II-induced mitochondrial dysfunction.

### 3.5. Vaspin Alleviates Ang-II-Induced Atrial Myocyte Dysfunction by Inducing Mitophagy

**Figure 5:**
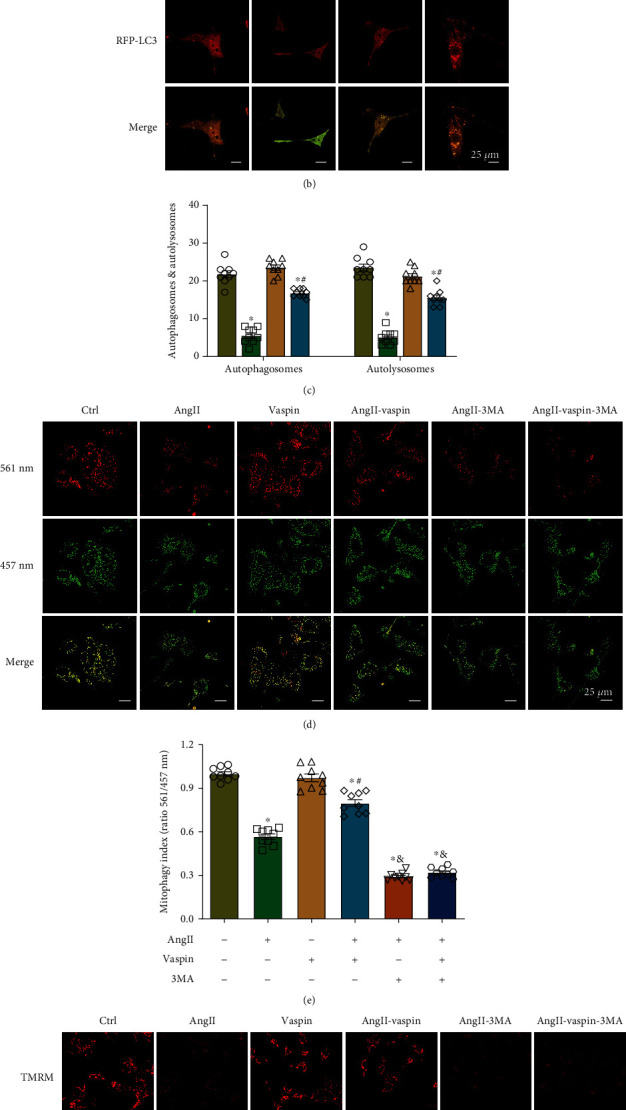
Vaspin induces mitophagy in Ang-II-treated atrial myocytes. (a) Representative immunoblots and quantification of Atg5, Beclin 1 in the whole cell lysates, and LC3II in the mitochondrial fraction of Ang-II-stimulated HL-1 cells, *n* = 6/group. (b–c) Representative images and quantification of mRFP-GFP-LC3 puncta, scale bar = 25 *μ*m. (d–e) Representative images and quantification of mito-Keima, scale bar = 25 *μ*m. (f–g) Representative images and quantification of TMRM staining, scale bar = 25 *μ*m; *n* = 9–12 fields/group, data from three independent experiments (panels b–g). Data are presented as the mean ± SEM, ^∗^*p* < 0.05*vs*. control group, ^#^*p* < 0.05*vs*. Ang-II group, ^&^*p* < 0.05*vs.* Ang-II-vaspin group.

To investigate the mechanisms underlying the beneficial effects of vaspin in AF, we examined mitophagy in HL-1 cells. Our results indicated that mitophagy was reduced in the Ang-II-stimulated groups, as demonstrated by the decreased levels of Atg5 and Beclin1, while vaspin treatment significantly attenuated this effect. Next, we evaluated the levels of mitophagy markers, and our results demonstrated that LC3II levels in the mitochondrial fraction were suppressed; however, vaspin administration reversed this effect (Figure 5(a)). To monitor autophagy and mitophagy in live cells, atrial cells were infected with mRFP-GFP-LC3 and mito-Keima adenoviruses after exposure to vaspin. Our data showed a decreased number of autophagosomes (yellow) and autolysosomes (red) in the Ang-II-stimulated groups, and this effect was alleviated by vaspin treatment. At the same time, vaspin alone did not affect the number of autophagosomes and autolysosomes (Figures 5(b)–5(c)). Thus, these results indicated that vaspin treatment induced autophagy and accelerated the fusion of autophagosomes and lysosomes.

To confirm the effect of vaspin on mitophagy in Ang-II-stimulated HL-1 cells, we used the autophagy inhibitor 3-methyladenine (3-MA). Our data showed that the fluorescence intensity excited at 561 nm, where the bright puncta colocalized with lysosomes, suggesting that the mitochondria in the lysosome were much weaker in Ang-II-induced atrial myocytes expressing mito-Keima than that in the control groups. This effect was partially alleviated by vaspin treatment.

Keima fluorescence intensity was significantly reduced in cells treated with 3-MA compared to that in the Ang-II-vaspin groups (Figures 5(d)–5(e)). Furthermore, TMRM results showed that 3-MA reversed the beneficial effects of vaspin on Ang-II-induced mitochondrial dysfunction in atrial myocytes (Figures 5(f)–5(g)). Therefore, these findings suggested that enhanced mitophagy played a crucial role in vaspin-dependent atrial-protective effects.

### 3.6. Vaspin Induces Mitophagy *via* the ULK1/FUNDC1-Dependent Mechanism

**Figure 6:**
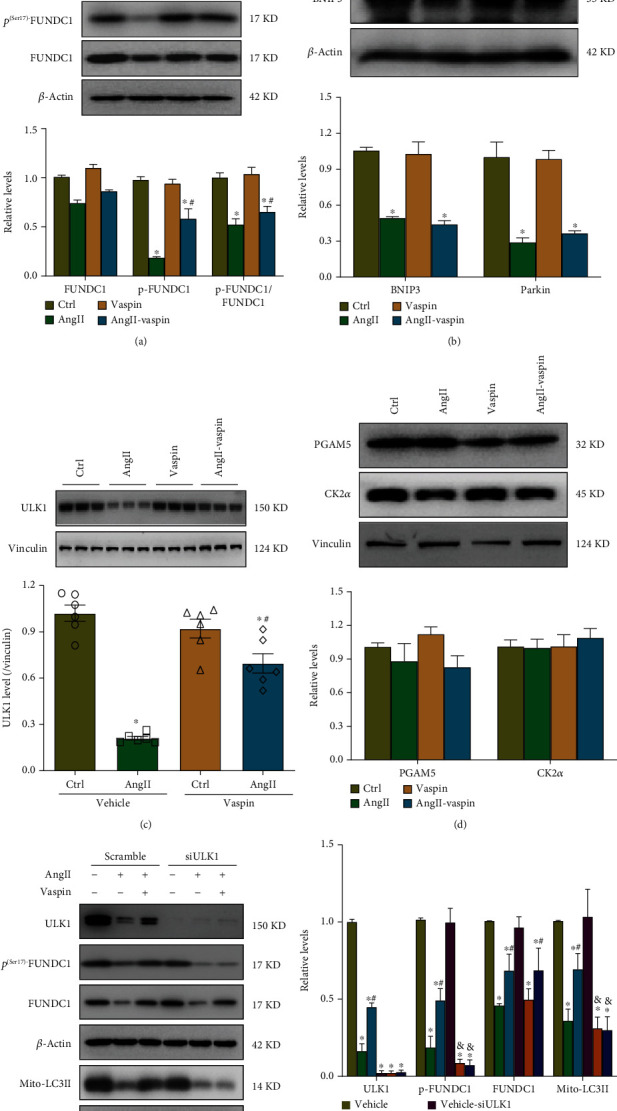
Vaspin induces mitophagy *via* a ULK1/FUNDC1-dependent mechanism. (a) Representative immunoblots and quantification of FUNDC1 and p^(Ser17)^-FUNDC1 in Ang-II-stimulated and vaspin-treated HL-1 cells. (B) Representative immunoblots and quantification of Parkin and BNIP3. (c) ULK1 in Ang-II-stimulated and vaspin-treated HL-1 cells. (d) Representative immunoblots and quantification of PGAM5 and CK2*α*. (e–f) ULK1, FUNDC1, p^(Ser17)^-FUNDC1, and LC3II in the mitochondrial fractions of Ang-II-stimulated and vaspin-treated ULK1-silenced HL-1 cells; *n* = 6 samples/group (panels a–f). Data are presented as the mean ± SEM, ^∗^*p* < 0.05*vs*. control group, ^#^*p* < 0.05*vs*. Ang-II group, ^&^*p* < 0.05*vs*. Ang-II-vaspin group.

FUNDC1, an integral mitochondrial outer membrane protein, has been reported to be an important mitophagy receptor [27]; therefore, we hypothesized that FUNDC1-mediated mitophagy was involved in the mechanism of vaspin-dependent protective action in cardiomyocytes. Our data demonstrated that FUNDC1 protein levels were reduced in Ang-II-stimulated HL-1 cardiomyocytes; however, vaspin treatment slightly ameliorated this effect (Figure 6(a)). We also evaluated the activity of other mitophagy pathways, including Parkin- and BNIP3-mediated mitophagy. Our data showed that Ang-II stimulation reduced the levels of Parkin and BNIP3, while vaspin treatment did not have any effect on their levels (Figure 6(b)).

Since the phosphorylation of FUNDC1 at Ser17 by ULK1 can enhance its binding affinity to LC3B and promote mitophagic activity [29], we measured p^(Ser17)^-FUNDC1 and ULK1 levels. Our results indicated that p^(Ser17)^-FUNDC1 and ULK1 protein levels were significantly decreased in HL-1 cardiomyocytes in response to Ang-II stimulation, while vaspin treatment partially restored these levels (Figure 6(c)). Moreover, the ratio of p^(Ser17)^-FUNDC1 to total FUNDC1 was significantly decreased after Ang-II stimulation, suggesting that Ser17 phosphorylation was involved in the development of atrial abnormalities. In addition, we evaluated other kinases known to phosphorylate FUNDC1, such as phosphoglycerate mutase family member 5 (PGAM5) and serine/threonine kinase casein kinase 2*α* (CK2*α*) [36, 37]. Our results showed that there was no effect on the levels of PGAM5 and CK2*α* in HL-1 cells upon Ang-II stimulation, with or without vaspin treatment (Figure 6(d)).

To further investigate whether ULK1 was involved in vaspin-dependent mitophagy, we silenced ULK1 in HL-1 cells using siRNA. Western blot assay showed that the levels of ULK1 were suppressed in ULK1 siRNA (siULK1)-transfected groups, compared to those in the control groups (Figures 6(e)–6(f)). Moreover, ULK1 knockdown significantly downregulated the levels of p^(Ser17)^-FUNDC1 and LC3II in the mitochondrial fraction following Ang-II stimulation. In addition, ULK1 knockdown nullified the protective effect of vaspin on p^(Ser17)^-FUNDC1 and LC3II in the mitochondrial fraction in HL-1 cardiomyocytes after Ang-II stimulation.

### 3.7. ULK1 Silencing Abolishes the Protective Effects of Vaspin on Ang-II-Induced Atrial Myocytes

**Figure 7:**
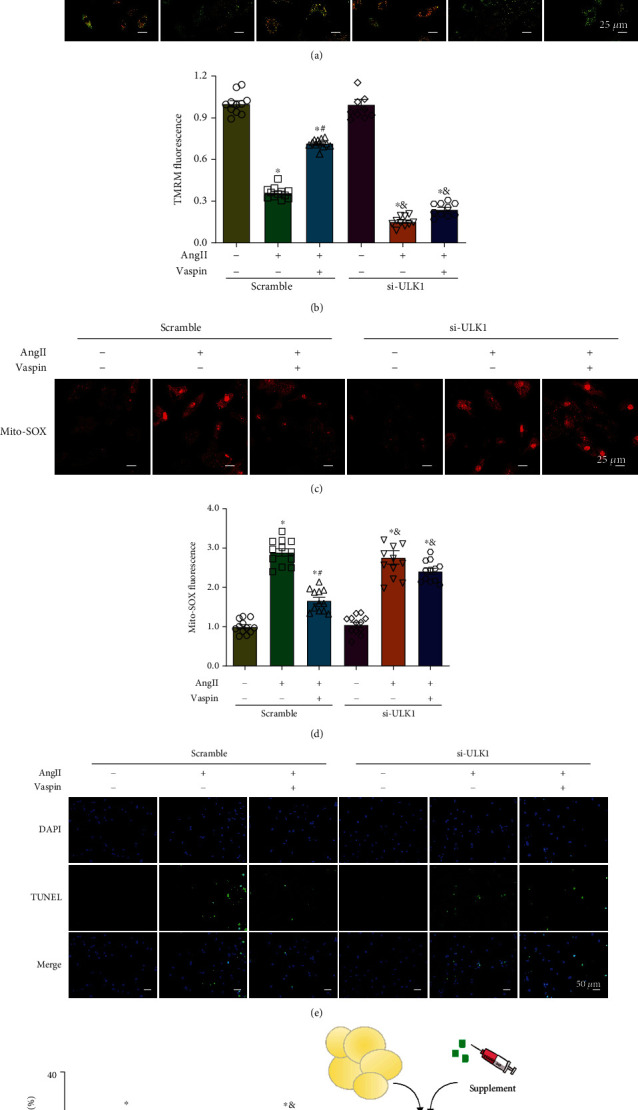
ULK1 silencing abolishes the protective effects of vaspin in Ang-II-induced atrial myocytes. Representative images and quantification of (a–b) MMP using TMRM staining, scale bar = 25 *μ*m. (c–d) Mitochondrial ROS accumulation using MitoSOX staining, scale bar = 25 *μ*m. (e–f) TUNEL analysis; *n* = 15–20 fields/group, data from three independent experiments (panels a–f). Data are presented as the mean ± SEM, ^∗^*p* < 0.05*vs*. control group, ^#^*p* < 0.05*vs*. Ang-II group, ^&^*p* < 0.05*vs*. Ang-II-vaspin group. (g) Schematic diagram depicting our hypothesis: vaspin treatment increased ULK1 levels and enhanced FUNDC1 phosphorylation at Ser17, thereby promoting mitophagy and mitigating mitochondrial damage in cardiomyocytes upon Ang-II stress.

To further examine the role of ULK1 in vaspin-regulated cardioprotective effects after Ang-II stimulation, we evaluated MMP, ROS generation, and apoptosis in HL-1 cells. Our results showed that in control groups, the fluorescence intensity of TMRM was significantly reduced after Ang-II stimulation, and this effect was mitigated by vaspin treatment. In contrast, TMRM intensity levels in the siULK1 groups were much lower than those in the control groups, and ULK1 silencing counteracted the protective effect of vaspin on Ang-II-induced mitochondrial dysfunction (Figures 7(a)–7(b)). MitoSOX staining results showed that in cells transfected with control siRNA, the level of MitoSOX was reduced after vaspin treatment in the Ang-II group, but this effect was nullified in the siULK1 groups (Figures 7(c)–7(d)). In addition, the results of TUNEL assay indicated that vaspin did not affect Ang-II-induced cardiomyocyte apoptosis in cells transfected with siULK1 (Figures 7(e)–7(f)). Therefore, our results suggested that vaspin could protect against mitochondrial damage in atrial abnormalities *via* ULK1/FUNDC1-dependent mitophagy.

## 4. Discussion

Here, we investigated the role of vaspin in AF and our findings indicated that vaspin was downregulated in the plasma of patients with AF. Furthermore, a lower vaspin concentration was associated with a higher risk of AF in patients with obesity. Our *in vitro* experiments demonstrated that vaspin treatment protected against Ang-II-induced atrial myocyte dysfunction, atrial fibrosis, and cardiomyocyte apoptosis. In addition, vaspin treatment increased ULK1 levels and enhanced Ser17 phosphorylation of FUNDC1, resulting in the induction of mitophagy and decrease of mitochondrial damage in cardiomyocytes in response to Ang-II (Figure 7(g)). To the best of our knowledge, this is the first study to investigate the effect of vaspin on FUNDC1-mediated mitophagy in an *in vitro* model of AF.

Vaspin, an adipokine derived from the epicardial adipose tissue, has been reported to participate in numerous diseases associated with abnormal metabolism, such as obesity, diabetes, and hypertension [38]. The majority of studies have focused on the effects of vaspin on insulin sensitivity^14^, while a limited number of contradictory studies attempted to investigate the mechanism underlying the beneficial effects of vaspin on cardiovascular diseases, especially AF [17, 39, 40]. Here, in this study, we observed that vaspin plasma concentrations were significantly reduced in patients with AF. Further analysis demonstrated that vaspin plasma levels could be an independent predictor of the occurrence of AF in obese patients with good specificity and sensitivity. *In vitro*, vaspin supplementation allowed the regular progression of atrial myocyte function and prevented mitochondrial damage and apoptosis, similar to its effects in vascular endothelial cells and coronary artery disease [41, 42]. Furthermore, our results also indicated that vaspin reversed Ang-II-induced mitophagy impairment and mitochondrial ROS production, confirming the previously reported effects of vaspin on mitochondrial function [16]. Collectively, our results suggest that, in addition to being a biomarker of AF in obese patients, vaspin could be a novel therapeutic target for AF.

Mitophagy, a type of selective autophagy, is a mitochondrial quality control mechanism that results in lysosomal degradation of dysfunctional mitochondria [25, 43]. Since mitochondria are vital intracellular organelles, impaired mitochondrial integrity and function can lead to the pathogenesis of various cardiovascular diseases [32, 33, 44]. It has been reported that autophagy has been affected in AF-induced cardiomyocytes; however, whether autophagic activity is upregulated or suppressed during the onset of AF remains debatable [45]. Our findings indicated that the levels of autophagy markers Atg5 and Beclin1 were initially reduced in Ang-II-treated HL-1 cells and this effect could be partially reversed by vaspin treatment. Next, we explored the regulation of mitophagy in Ang-II-induced atrial myocytes. Our results indicated that mito-LC3II expression levels were initially decreased upon Ang-II stimulation; however, these effects were ameliorated by vaspin treatment. The evaluation of mitophagic flux demonstrated that vaspin promoted the formation of autolysosomes and autophagosomes in atrial myocytes in response to Ang-II stimulation, which is consistent with previous reports indicating that vaspin mitigated myocardial ischemia/reperfusion injury by inducing autophagic flux [15]. Furthermore, mitochondrial activity and mitophagy were reduced after Ang-II stimulation, while autophagy inhibitor 3-MA reduced the mitochondrial function. These data are in line with previous findings indicating that mitochondrial injury induces atrial myocyte dysfunction after Ang-II stimulation [21]. Collectively, our results suggested that vaspin accelerated mitophagy flux to improve mitochondrial quality in response to Ang-II-induced atrial cardiomyocyte dysfunction.

It has been recently reported that adiponectin, an adipokine predominantly produced by adipose tissue and with pharmacological traits similar to vaspin, regulates mitophagy through Pink1/Parkin in mammalian cells [46]. In addition to Pink1/Parkin, mitophagy is also regulated by other cellular signaling molecules, including mitophagy receptors. For example, FUNDC1 is a mitophagy receptor anchored on the outer mitochondrial membrane [28]. Recent studies have demonstrated the beneficial properties of FUNDC1-mediated mitophagy in cardiac and cerebral ischemia-reperfusion injuries [36, 47]. Moreover, phosphorylation of FUNDC1 at distinct sites plays different roles in various pathological conditions [28]. PGAM5, a mitochondrial phosphatase, has been shown to dephosphorylate FUNDC1 at Ser13 and enhance its interaction with LC3B under hypoxic conditions [37]. At the same time, CK2*α* phosphorylates FUNDC1 at Ser13 to reverse the effect of PGAM5 on mitophagy, thereby playing a role in the development of cardiac IR injury [36]. Another study reported that FUNDC1 Ser17 phosphorylation mediated by ULK1 triggers FUNDC1-mitophagy activation and offers resistance to ischemic acute kidney injury [34]. Our data showed that the levels of ULK1, as well as and FUNDC1 Ser17 phosphorylation, and mitophagy were reduced in Ang-II-treated atrial myocytes, whereas vaspin supplementation reversed these effects and further protected against mitochondrial damage. In addition, ULK1 silencing reduced MMP and aggravated mitochondrial ROS accumulation and apoptosis in Ang-II-stimulated HL-1 cells. ULK1 silencing also eliminated the protective effect of vaspin against mitophagy impairment, mitochondrial dysfunction, and apoptosis in Ang-II-treated atrial myocytes, indicating that vaspin plays an essential role in ULK1-p^(Ser17)^-FUNDC1-mediated mitophagy in Ang II-induced atrial myocyte dysfunction and atrial fibrosis.

Our study has several limitations. This was a single-center, small sample-size, retrospective, nonrandomized control study. Additional prospective large sample-size multicenter clinical trials would be required to confirm the role of vaspin in AF in obese patients. Furthermore, our findings were obtained using an *in vitro* model system and should be validated in an *in vivo* model; unfortunately, there are no suitable animal models of AF. We observed that vaspin appeared to induce FUNDC1-mediated mitophagy; however, the exact mechanism of FUNDC1 phosphorylation and mitophagy activation in response to vaspin treatment is still not known.

In summary, our findings showed that vaspin increased mitophagy through ULK1/FUNDC1, thereby protecting against mitochondrial dysfunction and inhibiting atrial fibrosis. Although these results highlight the protective role of vaspin in promoting mitophagy and improving atrial myocyte aberrant function, further research is necessary to verify the effect of vaspin on the preservation of mitochondrial function and improvement of atrial fibrosis.

## Figures and Tables

**Table 1:** Clinical characteristics of patients in the SR and AF group.

Variables	SR group (*n* = 73)	AF group (*n* = 90)	*p*
Age (y)	63.0 ± 11.4	65.2 ± 11.1	0.533
Gender (%male)	39 (53.4)	56 (62.2)	0.259
BMI (kg/m^2^)	25.3 ± 1.5	25.9 ± 1.9	0.078
Smoking (%)	12 (16.4)	13 (14.4)	0.726
Drinking (%)	6 (8.2)	14 (15.6)	0.157
Hypertension (%)	35 (47.9)	50 (55.5)	0.335
Type II diabetes (%)	8 (10.9)	15 (16.7)	0.299
Hypercholesterolemia (%)	5 (6.8)	4 (4.4)	0.505
Echocardiography			
LVEF (%)	65.1 ± 52.6	65.1 ± 57.9	0.393
LAD (mm)	31.5 ± 4.4	39.5 ± 6.7	0.009
Laboratory			
BNP (pg/mL)	31.9 ± 42.3	201.8 ± 257.5	<0.001
TG (mmol/L)	1.3 ± 0.8	1.6 ± 1.0	0.365
TC (mmol/L)	4.3 ± 1.1	4.2 ± 1.1	0.903
LDL-c (mmol/L)	2.4 ± 0.9	2.5 ± 0.8	0.535
HDL-c (mmol/L)	1.2 ± 0.4	1.2 ± 0.3	0.144
Vaspin concentration (ng/mL)	8.4 ± 5.3	3.0 ± 3.5	<0.001
MMP2 concentration (ng/mL)	3.6 ± 3.1	8.4 ± 5.1	0.025
TGF-*β* concentration (ng/mL)	2.5 ± 2.1	4.2 ± 3.4	<0.001

Data expressed as mean ± SD. BMI: body mass index; LVEF: left ventricular ejection fraction; LAD: left atrium dimension; BNP: B-type natriuretic peptide; HDL-c: high density lipoprotein-cholesterol; LDL-c: low density lipoprotein-cholesterol; TG: triacylglycerol; TC: total cholesterol.

**Table 2:** Multivariate logistic analysis for predictors of patients with persistent atrial fibrillation.

	OR	95% CI	*p*
Gender	0.695	0.161-3.002	0.626
Smoking	1.842	0.204-16.653	0.587
Hypertension	1.429	0.334-6.108	0.63
Type 2 diabetes	0.524	0.095-2.895	0.458
Age(y)	1.002	0.946-1.062	0.939
LAD (mm)	1.327	0.906-1.769	0.019^∗^
TG (mmol/L)	1.074	0.426-2.710	0.879
BNP (pg/mL)	1.176	0.963-1.693	0.001^∗^
Vaspin (ng/mL)	1.437	1.178-1.753	<0.001^∗^
MMP2 (ng/mL)	1.288	0.801-1.683	0.008^∗^
TGF-*β* (ng/mL)	1.372	0.986-1.664	0.023^∗^

LAD: left atrium dimension; TG: triacylglycerol; BNP: B-type natriuretic peptide. ^∗^*p* < 0.05.

## Data Availability

The datasets used and/or analyzed supporting the findings of this study are available in this paper. Any raw data that support the findings of this study are available from the corresponding author upon reasonable request.
